# CNV-MEANN: A Neural Network and Mind Evolutionary Algorithm-Based Detection of Copy Number Variations From Next-Generation Sequencing Data

**DOI:** 10.3389/fgene.2021.700874

**Published:** 2021-08-16

**Authors:** Tihao Huang, Junqing Li, Baoxian Jia, Hongyan Sang

**Affiliations:** School of Computer Science and Technology, Liaocheng University, Liaocheng, China

**Keywords:** copy number variations, next-generation sequencing, mind evolutionary algorithm, multiple features, neural network

## Abstract

Copy number variation (CNV), is defined as repetitions or deletions of genomic segments of 1 Kb to 5 Mb, and is a major trigger for human disease. The high-throughput and low-cost characteristics of next-generation sequencing technology provide the possibility of the detection of CNVs in the whole genome, and also greatly improve the clinical practicability of next-generation sequencing (NGS) testing. However, current methods for the detection of CNVs are easily affected by sequencing and mapping errors, and uneven distribution of reads. In this paper, we propose an improved approach, CNV-MEANN, for the detection of CNVs, involving changing the structure of the neural network used in the MFCNV method. This method has three differences relative to the MFCNV method: (1) it utilizes a new feature, mapping quality, to replace two features in MFCNV, (2) it considers the influence of the loss categories of CNV on disease prediction, and refines the output structure, and (3) it uses a mind evolutionary algorithm to optimize the backpropagation (neural network) neural network model, and calculates individual scores for each genome bin to predict CNVs. Using both simulated and real datasets, we tested the performance of CNV-MEANN and compared its performance with those of seven widely used CNV detection methods. Experimental results demonstrated that the CNV-MEANN approach outperformed other methods with respect to sensitivity, precision, and F1-score. The proposed method was able to detect many CNVs that other approaches could not, and it reduced the boundary bias. CNV-MEANN is expected to be an effective method for the analysis of changes in CNVs in the genome.

## 1. Introduction

Copy number variations (CNVs) (Freeman et al., 2006; Redon et al., 2006) are important genomic structural variations, which are widespread in the human genome, and cause a variety of complex diseases, such as Crohn's disease (Fellermann et al., 2006; Aldhous et al., 2010), ankylosing spondylitis (Wang et al., 2013), Alzheimer's disease (Brouwers et al., 2012), and autism (Sebat et al., 2007). The accurate detection of CNVs in the human genome is an important step in the analysis of disease. Neither array comparative genomic hybridization (aCGH) (Peiffer et al., 2006) nor single-nucleotide polymorphism (SNP) (Park, 2008) microarrays enable an accurate determination of gene expression patterns via CNVs. However, next-generation sequencing (NGS) (Ansorge, 2009) techniques have introduced new possibilities for the accurate identification of CNVs with highly efficiency, low cost, and high-throughput (Tan et al., 2014).

In recent years, NGS-based methods have been employed for the identification of CNVs in the human genome. The bioinformatics tools used for detection of CNVs use four major approaches: paired-end mapping (PEM) (Shrestha and Frith, 2013), split-reads (Nguyen et al., 2016), sequence assembly (Nijkamp et al., 2012), and read depth (RD) (Yoon et al., 2009). Paired-end mapping uses many paired-end reads to detect CNV. For instance, PEMer (Korbel et al., 2009) detects CNVs using a simulation-based error model. CommonLAW (Hormozdiari et al., 2011) uses maximum parsimony analysis and aligns multiple samples to identify structural variants (SVs). GASV (Sindi et al., 2009) employs a geometric model for the detection of SVs. Pindel (Ye et al., 2009) detects break points and insertions using a pattern growth approach. There are several other PEM-based methods, including BreakDancer (Chen et al., 2009), Spanner (Mills et al., 2011), VariationHunter (Hormozdiari et al., 2010), SoftSV (Bartenhagen and Dugas, 2016), and PECC (Li et al., 2017). The PEM-based approaches can detect shorter CNVs, but are unable to identify CNVs in low coverage data.

The second category is based on split-reads, which divides the sequence into parts of equal size and aligns them to multiple locations of the reference genome. AGE (Abyzov and Gerstein, 2011) uses optimal alignments with gap excision to determine the breakpoints of genomic structural variants. SRIC (Zhang et al., 2011) calibrates all calls with realistic error models. Additional of split-read based approaches include SLOPE (Abel et al., 2010), SV-BET (Alzaid and Badr, 2016), and LAMSA (Liu et al., 2017).

The third category is an approach based on sequence assembly, which can detect CNVs by comparing the reference sequence and the assembled genome sequence. Magnolya (Nijkamp et al., 2012) utilizes a Poisson mixture model for the detection of CNVs from NGS data. Cortex (Iqbal et al., 2012) uses colored de-Bruijn graphs for identifying and genotyping gene mutation. Other methods based on sequence assembly include NOVOPlasty (Dierckxsens et al., 2017), MECAT (Xiao et al., 2017), Recycler (Rozov et al., 2017).

The last category is the read-depth-based approach, which is the most frequently used, and most effective method. The RD-based method detects CNVs by analyzing differences of read-depth signal in genomic locations (Klambauer et al., 2012). G-CNV (Manconi et al., 2015) is able to remove duplicated read sequences and normalize read-depth signal using graphics processing units. CNV-seq (Xie and Tammi, 2009) detects CNVs using shotgun sequencing. CNAseg (Ivakhno et al., 2010) implements a segmentation of the read counts (RC) using a Hidden Markov Model (HMM) to detects CNVs. Earlier RD-based approaches focused solely on read-depth signal. CNVnator (Abyzov et al., 2011) combines the mean-shift approach with correction of GC-content bias to expanded the range of CNVs found. BIC-seq (Xi et al., 2011) can discover CNVs via minimizing the Bayesian information criterion. ReadDepth (Miller et al., 2011) explains the overdispersion of genetic data by a robust statistical model. Control-FREEC (Boeva et al., 2012) constructs a profile that includes copy number and B-allele frequency for detecting CNVs. CNAnorm (Gusnanto et al., 2012) uses estimates of the mean to determine the underlying ploidy and the pollution level of normal cells. However, most of the abovementioned approaches can only detect CNVs characterizing distinct.

Many investigators have proposed CNV detection algorithms that consider RD signal plus other factors. For instance, m-HMM (Wang et al., 2014) uses the Expectation-Maximization (EM) algorithm to measure the parameters in the HMM, with their emission probabilities. CNV-RF (Onsongo et al., 2016) employs a random forest with a machine learning algorithm to find CNVs. CoNVaDING (Johansson et al., 2016) discovers CNVs busing a rigorous quality control standard that eliminates or marks low-quality exons. The seqCNV (Chen et al., 2017) method utilizes the maximum penalized likelihood estimation model to determine the CNV boundaries. ICopyDAV (Dharanipragada et al., 2018) enables to discovering the discovery of CNVs via a total variation minimization and circular binary segmentation algorithm. CONDEL (Yuan et al., 2020a) adopts a Bayesian approach with statistical mixture models to predict the status of the copy number. ModSaRa2 (Xiao et al., 2019) method uses a normal mean-based model in a screening and ranking algorithm for the detection of CNVs. BagGMM (Li et al., 2019b) utilizes a segmentation method involving “large bin and then small bins” to identify the boundaries of CNVs. CNV_IFTV (Yuan et al., 2019b) calculates an abnormal score for each genome bin using a set of isolation trees, and adopts a total variation model to flowing neighboring bins for detecting CNVs. SM-RCNV (Li et al., 2019a) combines the frequency of aberrance at one position across whole genomes and the correlation between successive positions for detecting CNVs. CNV-LOF (Yuan et al., 2019a) allocates an outlier factor to each genome bin, and uses a boxplot program box plot to distinguish CNVs. AITAC (Yuan et al., 2020b) creates a nonlinear model using an exhaustive search strategy to correlate observed and expected RDs. DINTD (Dong et al., 2020) adopts a spatial clustering algorithm based on density to discover the tandem duplication regions. CRSCNV (Liu et al., 2020a) uses a statistical model based on cross to measure the essentiality of genome bins. DCC (Yuan et al., 2018) utilizes a sliding bin to measure a relevant index of each genome segment for detecting CNVs. RKDOSCNV (Liu et al., 2020b) employs a kernel density estimation algorithm to calculate the partial kernel density distribution of each bin, and finds the CNVs by selecting an appropriate threshold. IndivCNV (Chen and Yuan, 2020) detects individual CNVs by hierarchical matrix energy spectrum. The features of existing approaches are shown in Table 1.

**Table 1:** Comparison of existing methods.

**Methods**	**Signals**	**Analysis samples**	**Data input**	**Language and interface**
DCC	RC	Single	BAM	Python
RKDOSCNV	RD, GC	Single	BAM	Python, R
DINTD	RD, MQ	Matched	BAM	Python
CNV-LOF	RC	Single	RC	Python, R
BagGMM	RC	Matched	BAM	MATLAB
SM-RCNV	RD	Single	RD	Java, R
MFCNV	RD, GC	Single	BAM	Python, MATLAB
CoNVaDING	RD	Matched	BAM, BED	Perl
CNV-RF	RD	Matched	BAM	Perl, R
CNAnorm	RC	Single	BAM	Perl, R
CNVnator	RD, SNP	Single	BAM	Python, Perl

*MQ, mapping quality file; Single, single sample analysis with normal matching samples; Matched, tumor-normal matched samples; CLI, command-line interface*.

The several approaches above rely on RD and other factors, reducing the effects of an uneven distribution of RC, to improve the sensitivity and specificity. However, these methods overlook the influence of linked effects of multiple features. And some CNVs that were similar to normal copy numbers not are identified due to the impacts of GC-content bias and sequencing errors. Artificial neural networks have powerful nonlinear mapping capabilities, which can address the linked effects of multiple features. MFCNV (Zhao et al., 2020) detects CNVs based on multiple features and a back-propagation neural network classifier. However, neural networks suffer from some limitations, such as slow convergence velocity, relapse into local optima, and premature convergence. These disadvantages reduce the generalization properties of neural networks.

Based on the above research, we propose an improved method called CNV-MEANN (CNV detection of neural network based on mind evolutionary algorithm). The proposed method consists of five steps: eigenvalue calculation and normalization, determination of neural network structure, calculation of individual scores by backpropagation neural networks using a mind evolutionary algorithm, training of the neural network, and prediction of the actual CNVs by the neural network. The major contributions of the presented method are as follows: (1) a feature called mapping quality is considered, which represents whether the mapping position of reads can be trusted, (2) the categories of CNVs are distinguished in great detail, including normal, gain, hemi_loss, and homo_loss, which helps to accurately distinguish the types of diseases, and (3) the network model parameters are optimized and the individual scores are calculated using a mind evolutionary algorithm, increasing the generalization capability of the network model, which further improves the sensitivity and precision of detection, to effectively identify CNVs without obvious characteristics.

The rest of this paper is organized as follows: section 2 introduces the workflow of CNV-MEANN, and the underlying principles and calculations. In section 3, some simulation datasets are applied to assess the performance of our method and several representative methods. Then, the proposed method is verified using some real sequencing samples. Finally, in section 4, a brief discussion and summary of the proposed method are provided, as well as outlines for future research.

## 2. Methods

### 2.1. Workflow of CNV-MEANN

CNV-MEANN is an RD-based method, and is designed to accurately detect CNVs using NGS data. At present, very few methods consider the effect of mapping quality. The original data is mapping to the reference genome to obtain the location information of the reads. Three features—GC-content, RD, and mapping quality—are integrated into the genome sequence analysis. The three factors interact in training a neural network. To circumvent the shortcomings of the network model, a mind evolutionary algorithm is adopted to optimize this model, enhance its generalization ability, and improve its performance. The individual scores are calculated by the mind evolutionary algorithm. CNV-MEANN greatly improves the sensitivity of detection of CNVs which do not have noticeable features.

**Figure 1:**
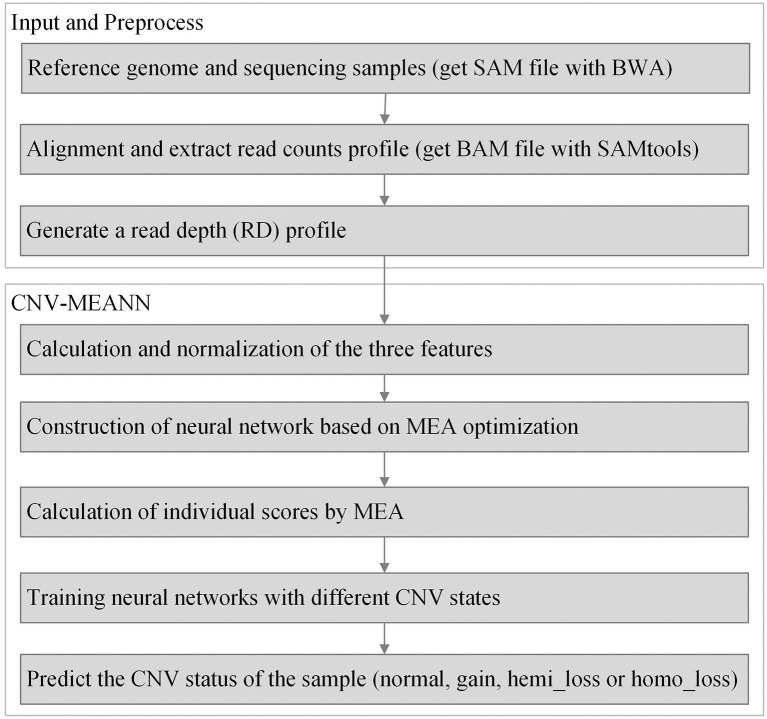
Workflow of the CNV-MEANN method for the detection of CNVs from NGS data.

The workflow of the CNV-MEANN method is depicted in Figure 1. First, a reference genome and a sequencing sample are used as initial input to CNV-MEANN. Then, CNV-MEANN pre-processes the sequencing input data. Finally, CNV-MEANN executes five main steps to achieve the identification of CNVs: (1) calculation and normalization of three features related to CNVs, (2) construction of the neural network model based on a mind evolutionary algorithm and using the three features, (3) calculation of individual scores by the mind evolutionary algorithm, (4) training of the neural network using marked CNVs acquired from simulation datasets and real sequencing samples, and (5) prediction of CNVs by the trained neural network model based on a mind evolutionary algorithm, where the output is the type of the CNVs (normal, gain, hemi_loss, or homo_loss) for each genome bin. The codes of the CNV-MEANN method are freely available at the website https://github.com/huangtihao/CNV-MEANN.git. In the following sections the five steps are illustrated in detail.

### 2.2. Data Preprocessing and Quantification of Eigenvalues

In CNV-MEANN, one of the alignment algorithms, BWA (Li and Durbin, 2009), is used to process a reference genome file (FASTA) and a sequencing sample file (FASTQ), to generate alignment files formatted in BAM. The RC profiles are acquired from the BAM file using the SAMtools (Li et al., 2009) software. The values of the RD signal are calculated from the RC profiles. Each RC profile is divided into successive and non-overlapping equally sized bins for computational convenience in calculating the RD signal. The default bin size is 1,000 bp, and can be set to other appropriate values by the users. The RD signal value is the mean value of the RC in each bin. GC-content bias is defined as high or low GC-content due to normal cell contamination during sequencing. Noise appears in the RD signal because of GC-content bias, and many methods calculate a pre-correction to overcome this bias. These approaches can deal with some specific scenarios, and the influence of GC-content bias is reduced, but they do not produce significant results in most clinical scenarios. Therefore, unlike the traditional approach, CNV-MEANN takes GC-content as one of the features for creating the input feature vector. The types of CNVs in the genome bin can be distinguished by mapping quality. If a read can be mapped to multiple locations, then the read will have a lower mapping quality. Mapping quality is also included in the analysis of the genome bins. Each genome bin is represented by a triple containing three factors, and it quantified as shown in Formula (1):

(1) $$\begin{array}{r} B_{i}=\left(R_{i},G_{i},Q_{i}\right) \end{array}$$

where *R*_*i*_ represents the RD value of the *i*-th bin, *Q*_*i*_ represents the mapping quality of the *i*-th bin and *G*_*i*_ represents the GC-content of the *i*-th bin. *B*_*i*_ is the eigenvector of the *i*-th genome bin. Since the proposed algorithm inputs triples, we combine the label column with triples and use a quadruple, *D*_m*4_ = (*g, q, r, L*), to represent the training data. Where *m* represents the number of bins to be used for training, *g*, *q*, and *r* represent the column vectors that the values of the three types of features, and *L* represents the column vectors of the label of bins. The values of *L* are 0, 1, 2, 3, which represent normal, gain, hemi_loss, and homo_loss, respectively.

Each feature has a different range of values. The value of *Q*_*i*_ is a few tens, while the value of *G*_*i*_ is between 0 and 1. We normalize the feature values using the min-max normalization method. Normalization of eigenvalues ensures they are invariant against scaling and magnification. Taking base quality as an example, normalization is shown in Formula (2):

(2) $$\begin{array}{r} Q=\frac{Q_{i}-Q_{\min}}{Q_{\max}-Q_{\min}} \end{array}$$

where *Q*_max_ and *Q*_min_ are calculated as the max and min of all bins in all RC profiles. Since normalization is for the mapping qualities of all bins, so *Q*_max_ and *Q*_min_ are calculated as max and min for all bins in all RC profiles.

### 2.3. Construction of a Neural Network

**Figure 2:**
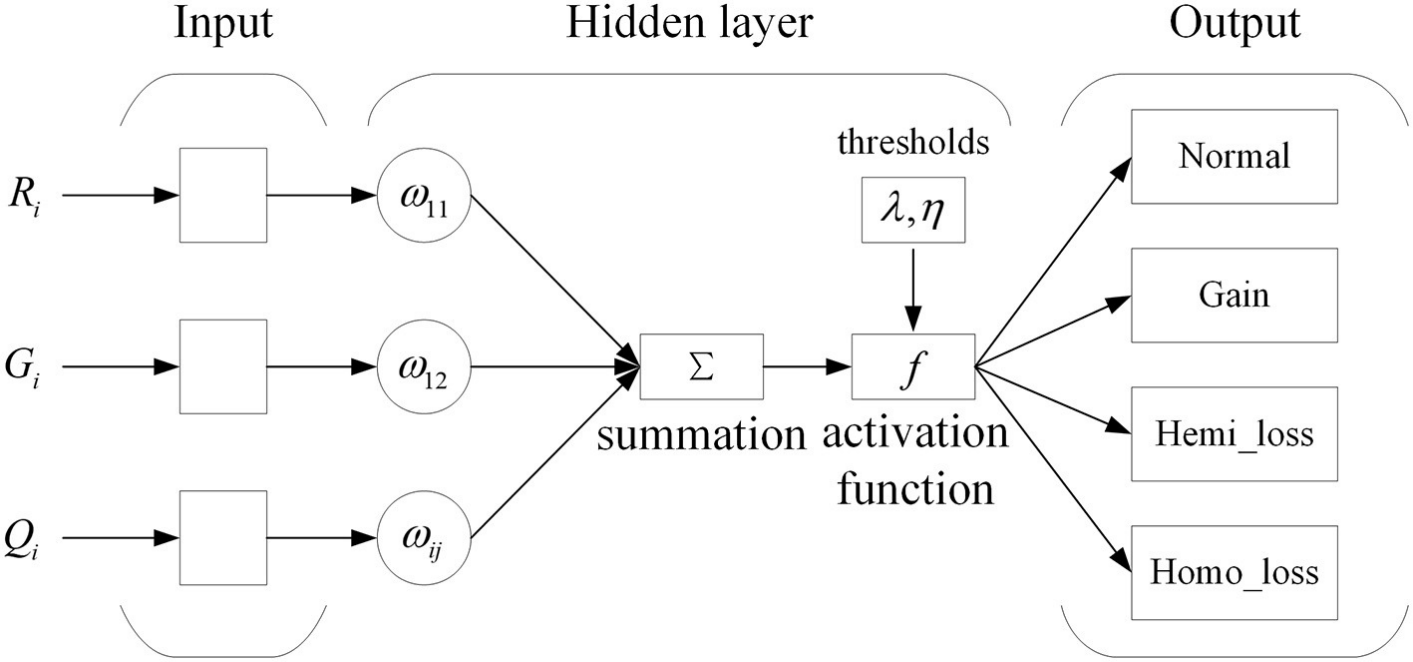
Structure of a neural network.

Based on the expression of each bin with an eigenvector *B*_*i*_ = (*R*_*i*_, *G*_*i*_, *Q*_*i*_), a network model using the three features for the prediction of CNVs is established. The neural network is the commonly utilized multilayer feedforward neural network trained using the backpropagation of errors algorithm. The neural network has been widely applied in pattern recognition and quality evaluation due to its excellent capability for nonlinear mapping and its flexible network structure. The training of a neural network involves the forward spread of signal and the reverse spread of error. During the forward spread, information comes from the input layer, is processed in the hidden layer(s), and transmitted to the output layer. If output results are inconsistent with the desired output, the matrix consisting of weights between neurons is adjusted, and the errors are reduced. The topology of the networks used in this work is shown in Figure 2.

In this picture, ω_*ij*_ indicates the weights between the input and hidden layer. The input layer consists of three neurons, the hidden layer consists of 15 neurons, and the output layer consists of four neurons. The neurons of the input layer represent three features for each genome bin. The neurons of the hidden layer are established according to calculation Formula *h* = *n*+3*m* following extensive experiments into the optimal functioning of the proposed algorithm, where *n* denotes the number of neurons in the input layer, and *m* denotes the number of neurons in the output layer. The neurons of the output layer indicate the four possible types of copy number: normal, gain, hemi_loss, and homo_loss. The nonlinear activation function between the input and hidden layers is the sigmoid function, which is shown in Formula (3):

(3) $$\begin{array}{r} f\left(\omega^{T}B+\theta \right)=\frac{2}{1+e^{-2\left(\omega^{T}B+\theta \right)}}-1 \end{array}$$

where ω represents the matrix of weights between the input and hidden layers, *B* represents the eigenvector from the input layer, and θ represents the corresponding errors. The learning rate is an important parameter of a neural network, and is closely related to convergence. The value of the learning rate is set at a default value of 0.1, which is the same as the MFCNV (Zhao et al., 2020).

### 2.4. Calculation of the Individual Scores by the Mind Evolutionary Algorithm

**Figure 3:**
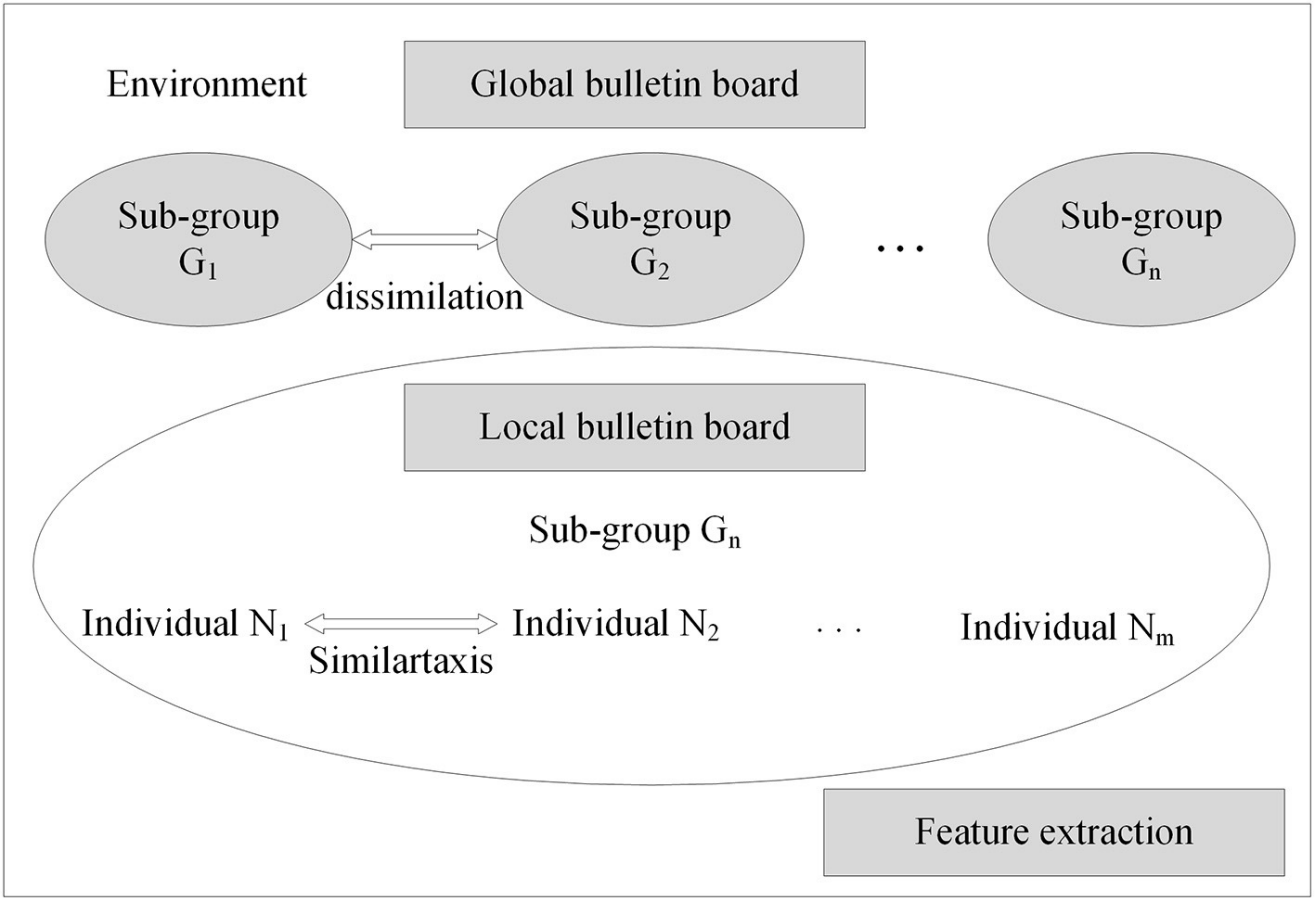
Structure of mind evolutionary algorithm.

With the neural network structure, a mind evolutionary algorithm is utilized to optimize the weights and thresholds of this network. The mind evolutionary algorithm and the evolutionary process of human thinking are very similar. The mind evolutionary algorithm not only retains the main concepts of the genetic algorithm, including group, evolution, and environment, but the algorithm is refined by the addition of two new concepts, similartaxis and dissimilation, proposed to simulate human thinking activity. The residual between the output produced and the expected output is lessened by the operation of similartaxis and dissimilation. Compared with the traditional optimization algorithms, the mind evolutionary algorithm speeds up the search process and enhances search efficiency. The system structure of the mind evolutionary algorithm is shown in Figure 3.

**Step 1:** Individual initialization. A population of individuals is generated randomly within the solution space, and the individuals are scored using the objective function. Superior individuals and temporary individuals are generated according to individual scores. The objective function is shown in Formula (4).

(4) $$\begin{array}{r} F=\frac{N}{\sum_{1}^{n}{\left(E_{i}-P_{i}\right)}^{2}} \end{array}$$

where *E* and *P* represent the values of the expected output and the produced output, respectively.

**Step 2:** Generation of superior subgroups. Random individuals are generated centered on superior individuals and temporary individuals, to obtain superior subgroups and temporary subgroups.

**Step 3:** Individual similartaxis. The operation of similartaxis is performed within each subgroup generated by the second step. After the subgroups are fully mature, the score of superior individuals in each subgroup is defined as the score of the corresponding subgroups, and the similartaxis operation is ended.

**Step 4:** Subgroup dissimilation. When the similartaxis operation is completed, the operation of dissimilation is performed between subgroups, and the scores of the subgroups are published on a global bulletin board until the process of replacement and abandonment between subgroups is completed.

**Step 5:** Generation of the best individual. After a predefined number of iterations, the individual with the maximum score is defined as the global optimal individual. The global optimal individual is defined as an individual composed of the optimal weight and threshold values optimized by the mind evolutionary algorithm (MEA). If the termination criterion is not fulfilled, the process is repeated from Step 3.

**Step 6:** The weights and threshold of the global optimal individual are allocated to a neural network. Then, the network model with the mind evolutionary algorithm is trained for the prediction of CNVs.

### 2.5. Training of the Neural Network Based on MEA

**Figure 4:**
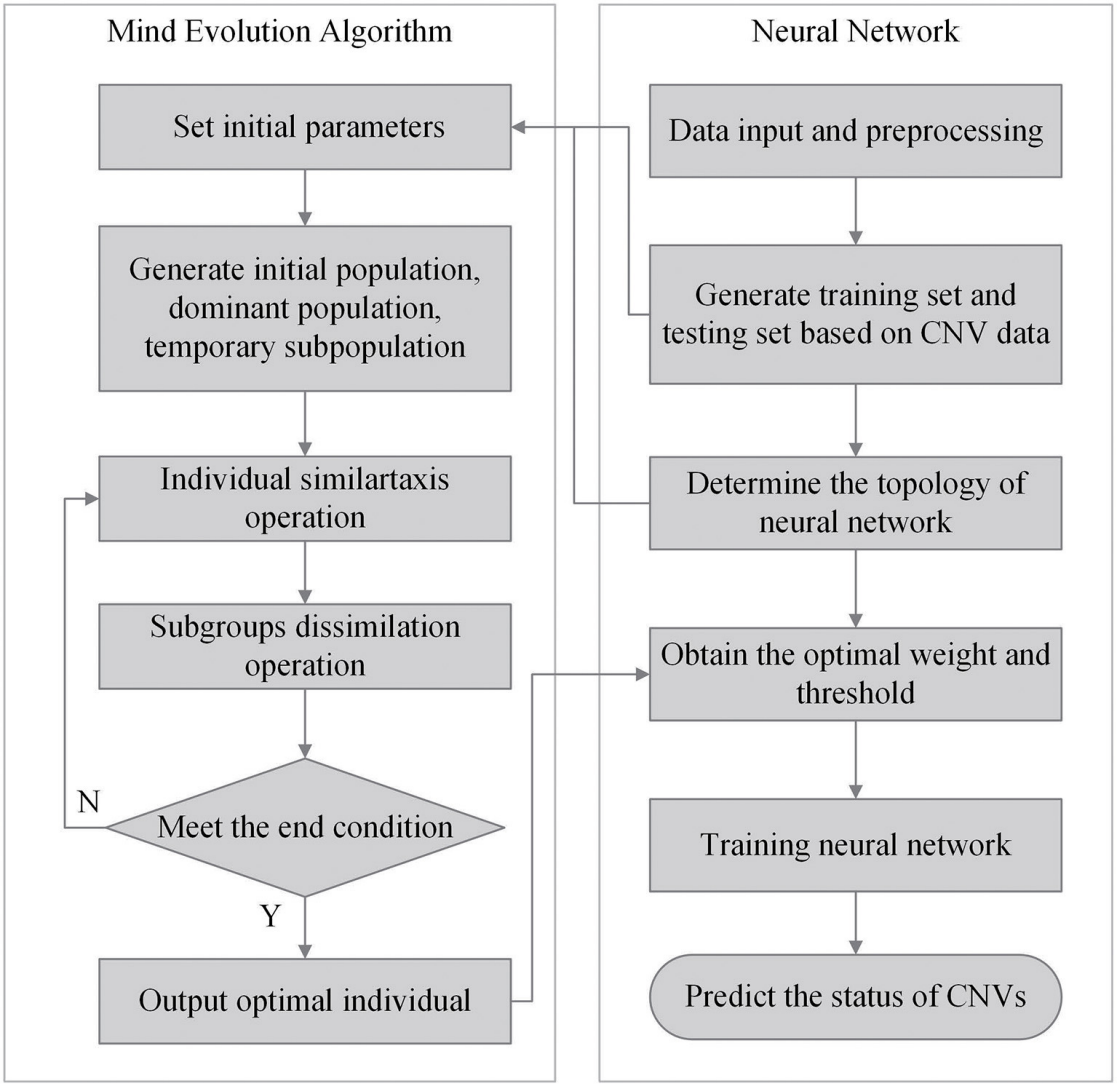
Flowchart of the network model with MEA.

The network model with MEA must be trained before being used. The specific steps of neural network are shown in Algorithm 1. First, the solution space is mapped to an encoding space, based on the topology of the neural network. Each code corresponds to a solution (that is, an individual). Then, the reciprocal of the average mean squared error from the training data is defined as the scoring function of each individual and population. The MEA is utilized to output the optimal individual after several iterations. Finally, the optimal individual is used as the initial weights and thresholds for the neural network, and used in training the network. A flowchart of the network model with MEA is shown in Algorithm 2 and Figure 4. The specific steps are as follows:

**Algorithm 1:** Procedure of neural network

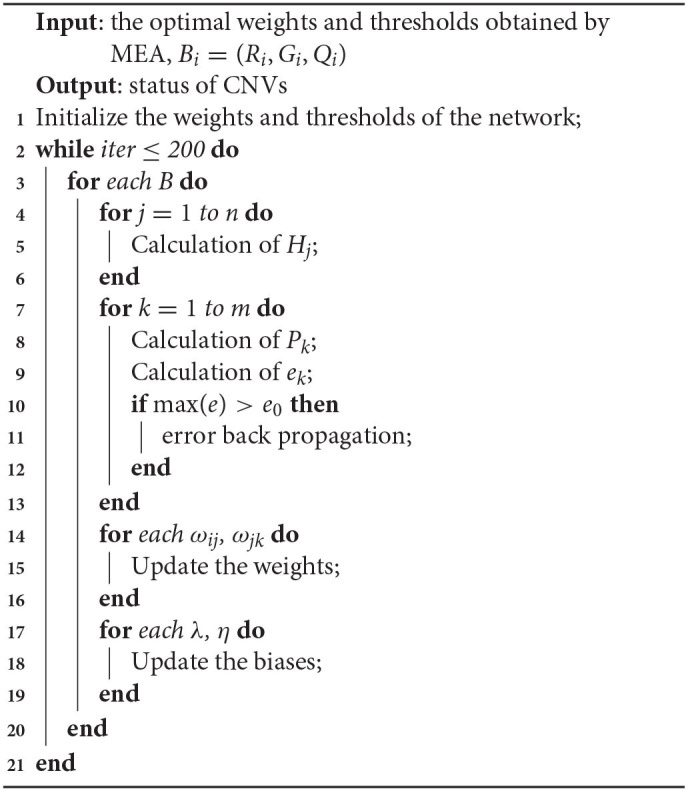

**Algorithm 2:** Procedure of MEANN

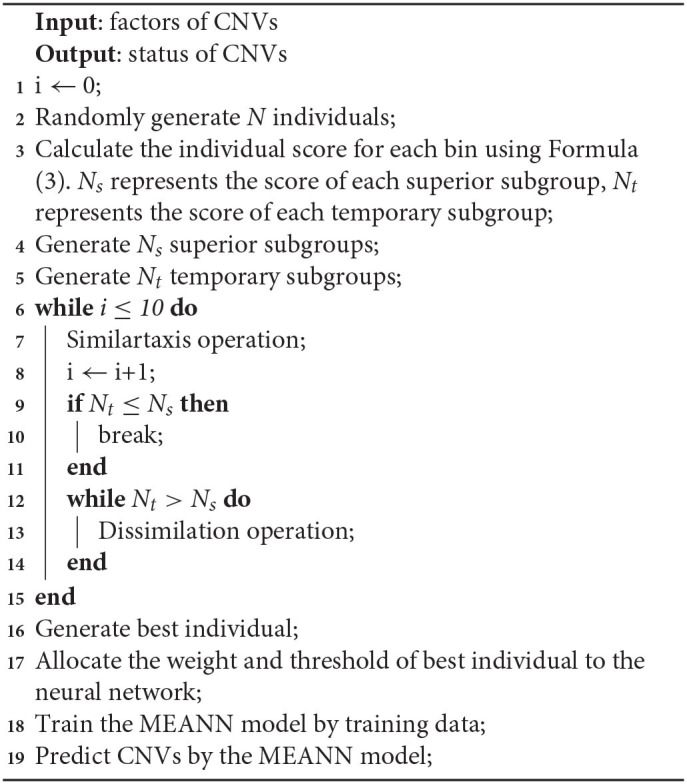

**Step 1:** Network initialization. The input vector is defined as *B*, the eigenvector of genome bins, and the output vector is defined as *P*, representing the four possible states of CNVs.

(5) $$\begin{array}{r} B=\left(b_{1},b_{2},\cdots \;,b_{n}\right)\\ P=\left(p_{1},p_{2},\cdots \;,p_{m}\right) \end{array}$$

**Step 2:** Calculation of the hidden layer. The activation function is defined as *f*.

(6) $$\begin{array}{r} H_{j}=f\left(\overset{n}{\underset{i=1}{\sum }}\omega_{ij}b_{i}-\lambda_{j}\right)\;1\leq j\leq h,1\leq i\leq n \end{array}$$

**Step 3:** Calculation of the output layer. The threshold is defined as η.

(7) $$\begin{array}{r} P_{k}=\overset{m}{\underset{j=1}{\sum }}H_{j}\omega_{jk}-\eta_{k}\;1\leq k\leq m \end{array}$$

**Step 4:** Calculation and verification of error. The error between the produced output and the expectation output is defined as *e*, and the maximum allowable error is defined as *e*_0_. If max(*e*)>*e*_0_, the parameters of the neural network are adjusted. The network is trained until the criteria have been fulfilled.

(8) $$\begin{array}{r} e_{k}=|E_{k}-P_{k}| \end{array}$$

**Step 5:** Update of the weights. The ω_*ij*_ and ω_*jk*_ are updated according to *e*, and the learning rate is defined as μ.

(9) $$\begin{array}{r} \omega_{ij}=\omega_{ij}+\mu H_{j}\left(1-H_{j}\right)b_{i}\overset{m}{\underset{k=1}{\sum }}\omega_{jk}e_{k} \end{array}$$

(10) $$\begin{array}{r} \omega_{jk}=\omega_{jk}+\mu H_{j}e_{k} \end{array}$$

**Step 6:** Update of the thresholds. The λ and η are updated according to *e*.

(11) $$\begin{array}{r} \lambda =\lambda +\mu H_{j}\left(1-H_{j}\right)\overset{m}{\underset{k=1}{\sum }}\omega_{jk}e_{k} \end{array}$$

(12) $$\begin{array}{r} \eta =\eta +e_{k} \end{array}$$

### 2.6. Prediction of CNVs

After training the neural network based on MEA, the prediction of CNVs can be made for the test data. For each genome bin, the output of the CNV-MEANN contains four values mapped from the activation function. The range of these values is (0,1), which represents the probability of each copy number status—normal, gain, hemi_loss, or homo_loss—for the bin. Normal is defined as the copy number without variation. Gain is defined as the repetition caused by homologous recombination between repetitive sequences on the same chromosome. Hemi_loss is defined as the deletion of copy number of a homologous chromosome. Homo_loss is defined as the simultaneous deletion of the copy number of two homologous chromosomes. The status of the candidate CNVs is then determined as the largest of the four probability values. For example, the candidate CNV is defined as a deletion if the probability of gain is larger than the probability of hemi_loss, homo_loss, and normal. Finally, subgroups are created using each candidate CNV as the center of a group, so that the actual CNVs are identified.

## 3. Results

### 3.1. Simulated Data Studies

Simulation experiments were used to evaluate the reliability and accuracy of our method. The parameters of the CNV-MEANN method are shown in Table 2. A comprehensive simulation approach, IntSIM (Yuan et al., 2017), from methods of analysis of genomic variation was chosen to generate simulated data. Chromosome 21 from the reference genomes was selected as input sequence of IntSIM. The range of tumor purity was set to 0.2, 0.3, and 0.4, the range of coverage depth was set to be 4x and 6x, and the different sequence datasets were generated using different configurations. Fifty samples were generated for each configuration, making 300 samples in total.

**Table 2:** Parameters of the CNV-MEANN method.

**Parameters**	**Scale**
Number of input layer	3
Number of hidden layer	15
Number of output layer	4
Weights	3*15+15*4 = 105
Thresholds	15+4 = 19
Population	200
Superior subgroups	5
Temporary subgroups	5
Subgroups	20

**Figure 5:**
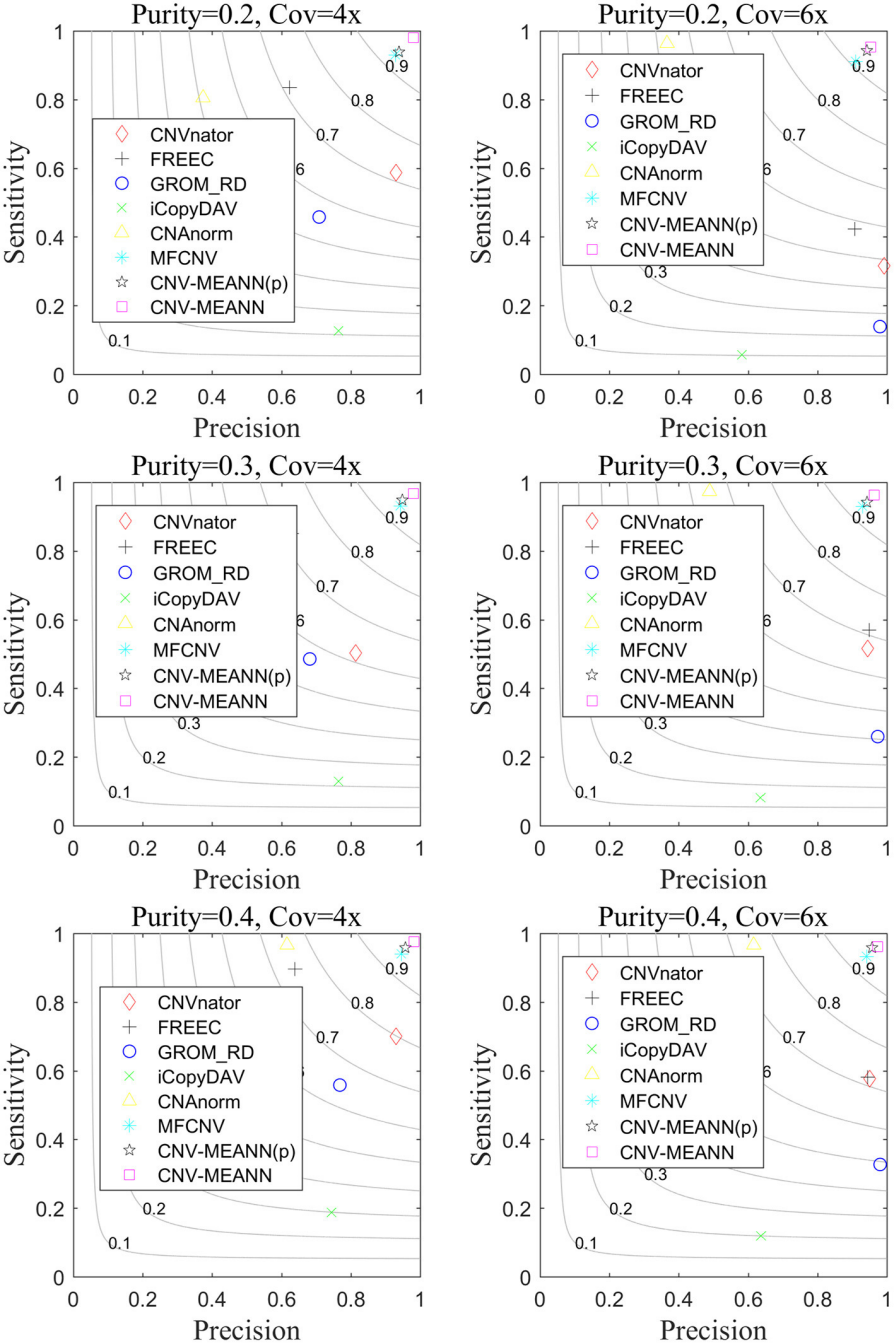
The performance comparison of the CNV-MEANN method and the eight same methods in the aspects of sensitivity, precision and F1-score on simulation datasets under six configurations.

Based on these simulated data, CNV-MEANN and six alternative methods, CNAnator, iCopyDAV, FREEC, CNAnorm, GROM_RD, MFCNV, were run. The default parameters were used to ensure a fair comparison. The comparison of the performances of the seven methods is shown in Figure 5. The sensitivity is defined as the ratio of the number of accurately identified CNVs to the totality of ground truth CNVs. The precision is the ratio of the number of accurately identified CNVs to the totality of all identified CNVs. The F1-score (gray curves in Figure 5) is the harmonic mean of sensitivity and precision. The values of each indicator represent the means of 50 samples from each configuration.

It can be seen from Figure 5 that the performance of the seven methods improves with increasing tumor purity. For example, the sensitivity of GROM_RD was about 0.45 at a tumor purity of 0.2 and a coverage depth of 4x, while it reached 0.56 at a tumor purity of 0.4 and a coverage depth of 4x. Of the methods, the lowest F1-score value was about 0.11 and the largest value was about 0.94 at a tumor purity of 0.2 and a coverage depth of 6x, while these methods achieved 0.20 and 0.96 at a tumor purity of 0.4 and a coverage depth of 6x.

CNVnator achieved the highest precision in one environment, GROM_RD achieved the highest precision in two environments, and CNV-MEANN achieved the highest precision in three environments. From the perspective of sensitivity, CNV-MEANN reached the highest value in four environments, CNAnorm achieved the highest value in two environments. From the perspective of F1-score, CNV-MEANN reached the highest value in all environments, and MFCNV had the second highest value. Thus, the performance of these methods can be ordered from highest to lowest as follows: CNV-MEANN, MFCNV, FREEC, CNAnorm, CNVnator, GROM_RD, and iCopyDAV.

Regarding each type of CNV, the proposed algorithm can also accurately detect. At a sample with tumor purity of 0.2 and coverage depth of 4x, the number of CNV of gain type, hemi_loss type, and homo_loss type is 166, 144, and 144, and the proposed algorithm identifies 159, 143, and 141, respectively. And at a sample with tumor purity of 0.3 and coverage depth of 6x, the proposed algorithm identifies 162, 136, and 131, respectively.

To discuss how much improvement due to the use of MEA. The prior algorithm of CNV-MEANN method which only uses MEA algorithm without introducing new features is named CNV-MEANN(p). The CNV-MEANN(p) method was compared with other algorithms, and the results were shown in Figure 5. The average F1-score of CNV-MEANN(p) is 0.946, while the average F1-score of MFCNV is 0.931, and the detection ability is improved by 0.015. The reason for this result is that MEA divides the population into superior subgroups and temporary subgroups. On this basis, the similartaxis and dissimilation operations are respectively explored and developed. These two operations coordinate with each other and maintain certain independence, which is convenient to improve the efficiency, respectively. Besides, MEA can remember multiple generations of evolutionary information, which can guide similartaxis and dissimilation in a favorable direction.

**Figure 6:**
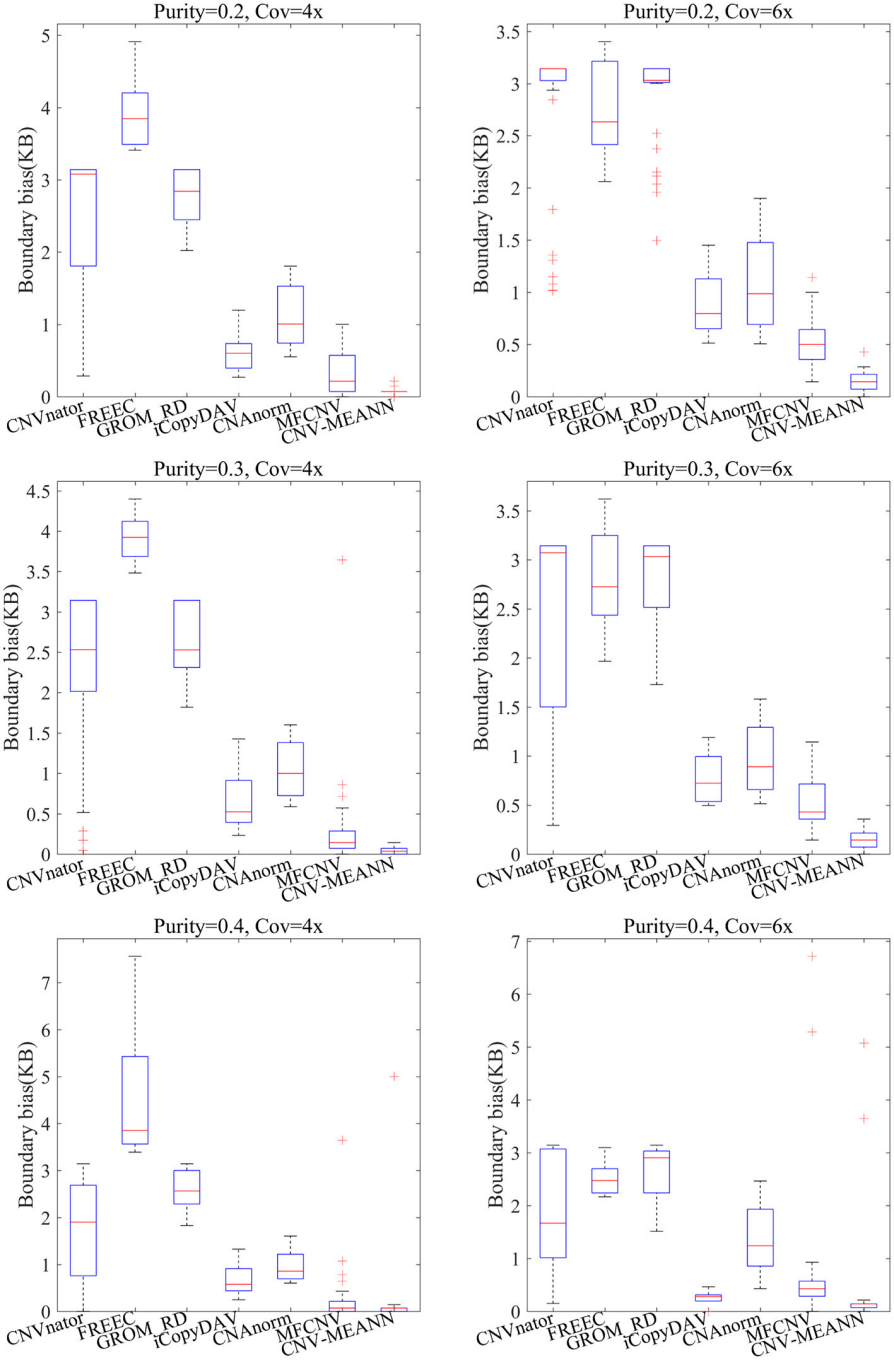
Comparisons of the boundary bias between CNV-MEANN and six peer methods.

The accuracy of boundary bias detection is also an important indicator of the reliability of the proposed method. The boundary bias is described as the bias between the mean number of base pairs of the identified CNVs and the ground truth CNVs in each simulation sample. The smaller the boundary bias, the higher the performance of the CNV detection method. The boundary bias of the seven methods is shown in Figure 6.

As can be seen from Figure 6, the boundary bias of the seven methods showed a downward trend with increase in tumor purity and coverage depth. In contrast, CNV-MEANN obtained the lowest value in five environments, and MFCNV obtained the lowest value in one environment. In the comparison of boundary bias, the performance of these methods can be ordered from highest to lowest as follows: CNV-MEANN, MFCNV, iCopyDAV, CNAnorm, CNVnator, GROM_RD, and FREEC. The main reason for the good boundary bias of CNV-MEANN is it detected the CNVs that are were not detected by other methods.

**Figure 7:**
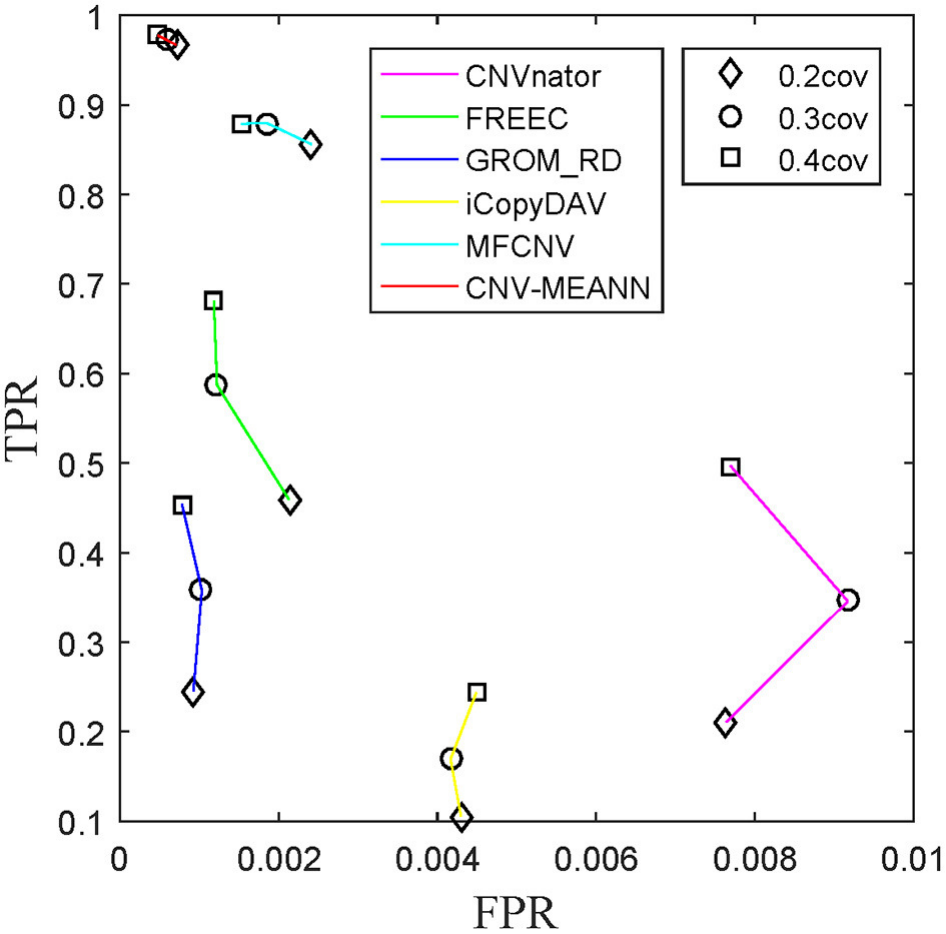
ROC plots on simulation datasets between CNV-MEANN and five peer methods.

ROC curves were also used to assess the performance of the detection methods. ROC curves are constructed by plotting the true positive rate (TPR) against the false positive rate (FPR). The curve with both higher values of TPR and lower values of FPR, the performance of the corresponding method is thought to be better. The TPR and the FPR for each tumor purity of the six methods were calculated and compared, and the results are presented in Figure 7. The CNV-MEANN method performed better than the other five methods in TPR, and performed slightly better than the GROM_RD method in FPR. The MFCNV method had a higher value of TPR and a lower value of FPR, and its performance was next to the MEANN method. In summary, CNV-MEANN had superior performance compared to the other methods, especially for low tumor purity and low coverage data.

There are three main reasons for the superior performance of CNV-MEANN: (1) it selected numerous samples used as the training data from data of different configurations, and the fault tolerance is improved, (2) it extracted a feature called mapping quality that better reflects the states of CNV, and considered the joint actions of multiple features by using the neural network, whereas other methods only consider the marginal effects of each feature, and (3) it used an MEA to optimizing the neural network, enhancing the robustness of the model.

### 3.2. Real Data Applications

To further investigate the effectiveness of CNV-MEANN, we applied it to three real sequencing samples from the CEU family of the 1,000 Genomes Project (http://www.internationalgenome.org/), ID numbers NA19238, NA19239, and NA19240. The CNV-MEANN method and the other six methods were used to detect CNVs on the chromosome 21 of each sample, and the results are presented in Table 3. For these real sequencing samples, the Database of Genomic Variants (DGV) (http://dgv.tcag.ca/) was used to obtain validated CNVs. These validated CNVs were used to measure the performance of CNV-MEANN and the other peer methods.

**Table 3:** Number of CNVs detected by CNV-MEANN and the six peer methods on real sequencing samples.

**Samples**	**CNVnator**	**GROM_RD**	**FREEC**	**iCopyDAV**	**ReadDepth**	**MFCNV**	**CNV-MEANN**
NA19238	252	0	222	26	220	233	275
NA19239	145	5	91	11	187	181	194
NA19240	109	9	88	8	211	183	153

**Figure 8:**
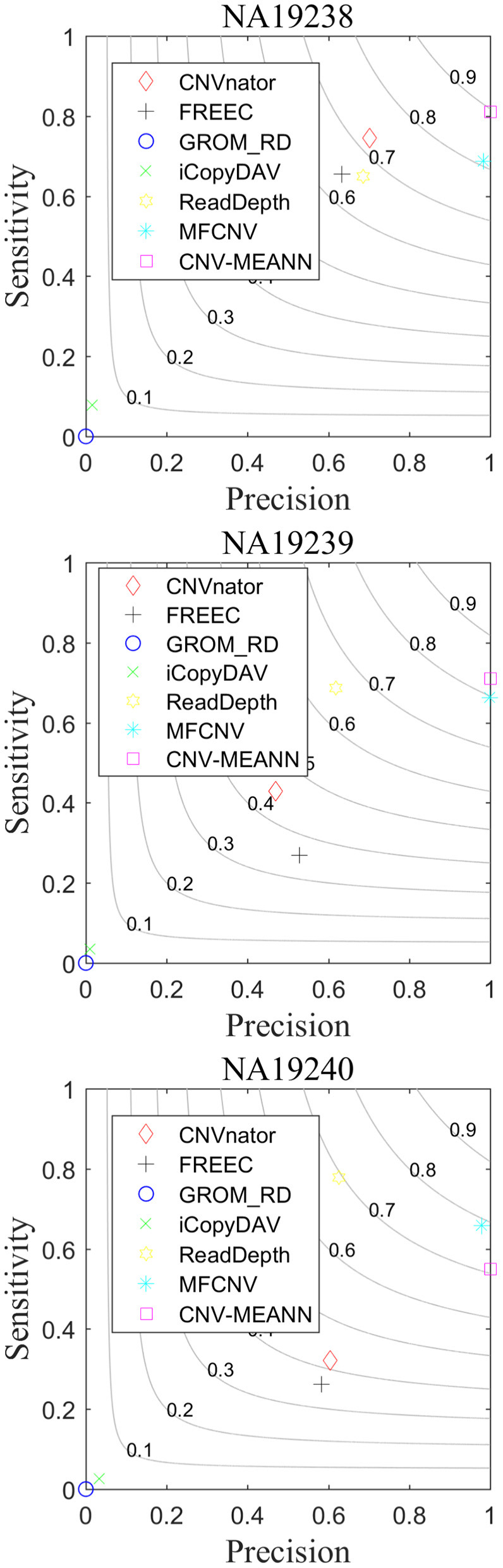
Performance comparison of the six methods on real sequencing samples.

As can be seen from Table 3, CNV-MEANN identified 275, 194, and 153 CNVs in these real sequencing samples. Compared with the other six methods, the number of CNVs detected by CNV-MEANN was less than that detected by MFCNV for the sample NA19240. The total number of CNVs detected by CNV-MEANN was 4.19% higher than that detected by MFCNV. This observation indicates that CNV-MEANN has a strong capacity for the detection of CNVs with non-obvious features. The comparisons of sensitivity, precision, and F1-score are shown in Figure 8.

As can be seen from Figure 8, CNV-MEANN had the highest sensitivity for two samples, while ReadDepth had the highest sensitivity for sample NA19240, followed by MFCNV and the other methods. With respect to precision, MFCNV had the highest value for sample NA19239, while CNV-MEANN had the highest values for the other two samples, followed by CNVnator and the other methods. From the perspective of the F1-score, CNV-MEANN had the highest values in two samples, a value of around 0.81 for three samples, followed by MFCNV and the other methods. Compared with the other six methods, CNV-MEANN achieved a balance between sensitivity and precision. The results demonstrate that CNV-MEANN performed well in a practical application.

## 4. Discussion and Conclusion

In research into intricate human diseases, the accurate detection of CNVs is essential for comprehensive analyses of genome sequence mutations. In this paper, an improved method for the detection of CNVs from NGS data, called CNV-MEANN, is presented. CNV-MEANN was developed based on the RD method, and integrates multiple features associated with CNVs. The algorithm includes a neural network model and utilizes an MEA to predict CNVs. Unlike traditional CNV detection methods, that only focus on the importance of the RD signal, our method not only considers the influence of multiple features, but also explores the impact of correlation between different features. Compared with extant methods, CNV-MEANN does not demand correction for GC-content bias, so it can elude the errors generated during this process. CNV-MEANN extracts and integrates three important features that are closely related to CNVs and genomic structure variation. It then uses the neural network to address the weight interactions of these features. CNV-MEANN uses datasets of various configurations to train the neural network algorithm, using training data with different grades of tumor purity and coverage depth to enhance the generalizability and adaptive capacity of the algorithm. Finally, CNV-MEANN employs an MEA to calculate the individual scores for the prediction of different types of CNVs.

Each of the three factors used in the algorithm are likely to have its own fringe effect, that affects the accuracy of CNV predictions. Fringe effect is defined as the effect of a single feature on the prediction of copy number variation. The CNV-MEANN method integrates the three features and takes them as the input to a neural network. During the training of the neural network, CNV-MEANN uses backpropagation of errors to update the weights and thresholds, which includes the fringe effect of each feature. The joint effect among the features also affects the prediction of CNVs. Because each of the features has a different range of values, the features are normalized before they are input into the neural network. Normalization scales the eigenvalues to the same level and balances the three features. To overcome the shortcomings of the network model, such as relapsing into local optima, the MEA is used to improve the predictive ability of the model. In the process of optimizing the neural network using the MEA, the eigenvectors score, composed of three eigenvalues, is calculated, and the individual with the highest score is regarded as the global optimal individual. Superior subgroups are generated centered on superior individuals. The superior subgroups contain many CNVs. Similartaxis is then performed within the superior subgroups. After the subgroup matures, the score of superior individuals (the eigenvector that is marked as CNV) in this subgroup is regarded as the score of the subgroup. After the operation of dissimilation is performed between subgroups, the unmarked CNVs have a probability of being marked as CNVs in each subgroup. Finally, the marked eigenvectors are employed as inputs to the neural network to predict CNVs.

To appraise the performance of CNV-MEANN, several comprehensive datasets of including different tumor purity and coverage depth were simulated. CNV-MEANN was tested for sensitivity, precision, and F1-score. CNV-MEANN had a better performance than six other competing methods. CNV-MEANN was validated using three real sequencing samples from different sequencing platforms, and the validated CNVs of the DGV were used to measure the performance of CNV-MEANN and the other peer methods. CNV-MEANN performed better than the other six methods in practical application. Therefore, CNV-MEANN is a reliable and effective tool for identifying CNVs from NGS data, especially for datasets with low tumor purity and low coverage.

With respect to future work, we plan to improve CNV-MEANN in the following four aspects. First, the estimation of tumor purity and ploidy will be researched and integrated into CNV-MEANN. This procedure will help to reduce the impact of normal genome pollution in the sequenced genomes, and provide more diagnostic information. Next, the application of CNV-MEANN to single-cell sequencing data will be expanded. Genome bins of single-cell sequencing data containing normal and distorted states will be used to train the CNV-MEANN algorithm, which will contribute to the discovery of new intercellular heterogeneity, and corresponding mutations. Then, simultaneous detection of SNV and CNV will be considered, as both tend to occur on the genome (Mao et al., 2021). Finally, an alternative neural network structure (Li et al., 2021) and a custom population size can be implemented, which can adapt to different sizes of sequencing data. Judging by its superior performance, CNV-MEANN can be applied to clinical diagnosis and improve the ability to predict CNVs.

## Data Availability Statement

The datasets presented in this study can be found in online repositories. The names of the repository/repositories and accession number(s) can be found at: https://www.ebi.ac.uk/dgva/, 19238, 19239, 19240.

## Author Contributions

TH participated in the design of algorithms and experiments. JL and HS participated in the design of the whole framework of detecting CNVs. TH and BJ participated in the analysis of the performance of the proposed method. JL and TH conceived the study and helped in editing the manuscript. All authors read the final manuscript and agreed on its submission.

## Conflict of Interest

The authors declare that the research was conducted in the absence of any commercial or financial relationships that could be construed as a potential conflict of interest.

## Publisher's Note

All claims expressed in this article are solely those of the authors and do not necessarily represent those of their affiliated organizations, or those of the publisher, the editors and the reviewers. Any product that may be evaluated in this article, or claim that may be made by its manufacturer, is not guaranteed or endorsed by the publisher.
